# Enhancing Athletic Performance: A Comprehensive Review on Kettlebell Training

**DOI:** 10.7759/cureus.53497

**Published:** 2024-02-03

**Authors:** Pratik R Jaiswal, Swapnil U Ramteke, Saylee Shedge

**Affiliations:** 1 Sports Physiotherapy, Ravi Nair Physiotherapy College, Datta Meghe Institute of Higher Education & Research, Wardha, IND

**Keywords:** dynamic balance, agility, multi-sports athletes, sports physiotherapy, exercise, kettlebell training

## Abstract

A kettlebell is a weight made of cast iron shaped like a ball with a handle. Commercial kettlebells are offered in capacities that vary from 3 pounds to 100 pounds (or more). The kettlebell has a variety of possible clinical applications, including dynamic flexibility exercises and power training. Players' efficacy during the game can be improved by developing their strongest potential prior to exercising and efficiently converting that strength to power as the event draws near. Strengthening has been recommended as an effective means to avoid injuries, build muscle strength, and enhance one's health in relation to performance in the game. This type of training focuses on the hip, thigh, core, and abdominal muscles to help with appropriate lower-limb alignment and the recruitment of muscle patterns. Kettlebell training is a flexible and useful strategy for improving players' performance in a variety of sports. It adds value to athlete training programs by enhancing strength, power, endurance, explosive power, and postural coordination.

## Introduction and background

Resistance training has been shown to improve strength, power, and endurance in a variety of ways. Since ancient times, people have utilized resistance training to improve their strength, power, endurance, and functional abilities [1]. Over the past 20 years, resistance training has become more and more popular as a form of exercise because it can enhance the performance of athletes by boosting muscular hypertrophy, power, speed, and endurance, as well as motor function, balance, and coordination [2]. Elite fitness groups and athletes frequently quickly adopt techniques that are employed by successful competitors or promoted by other performance-based organizations [3]. Sports success is influenced by an extensive array of complicated factors, including sociological, mental, and physical (generic and specialized) circumstances [4]. Increasing strength, power, and endurance is a constant demand in the fields of sports medicine, athletics, rehabilitation, and health. There is a constant trial and adoption of novel training techniques with differing levels of outcome evaluation [5]. Players can increase efficiency by using preconditioning training, which includes heavy resistance or ballistic actions, in the few moments before power-based workouts [6-10].

Athletes must build their maximal strength in workouts and then properly convert that strength to power as the competition draws near in order to succeed at their peak during matches [11]. In sports, lower-limb strength is crucial for improving balance and agility and for producing the power required for explosive actions. The major musculature at the lower limb and the gluteals, adductors, abductors, hamstrings, and quadriceps are crucial for movements needed for daily tasks or athletic endeavours. Lower-body strength is vital because it promotes quicker and more accurate footwork. The powerful lower body enhances speed when travelling around the court by enabling quick stops and direction changes. Additionally, by transmitting energy from the legs to the upper body, stronger lower-limb muscles enable the production of stronger smashes, such as serving in tennis and smashing in volleyball [12]. This type of exercise focuses on the hip, thigh, core, and abdominal muscles to help with proper lower-body alignment and the recruitment of muscle patterns. All this happens by enhancing strength and stability, improving joint alignment, balanced muscle development, core activation, improved flexibility, and range of motion. For younger players, it is especially crucial because injuries could have an adverse impact on their preparation, game performance, and athletic career [13].

**Figure 1:**
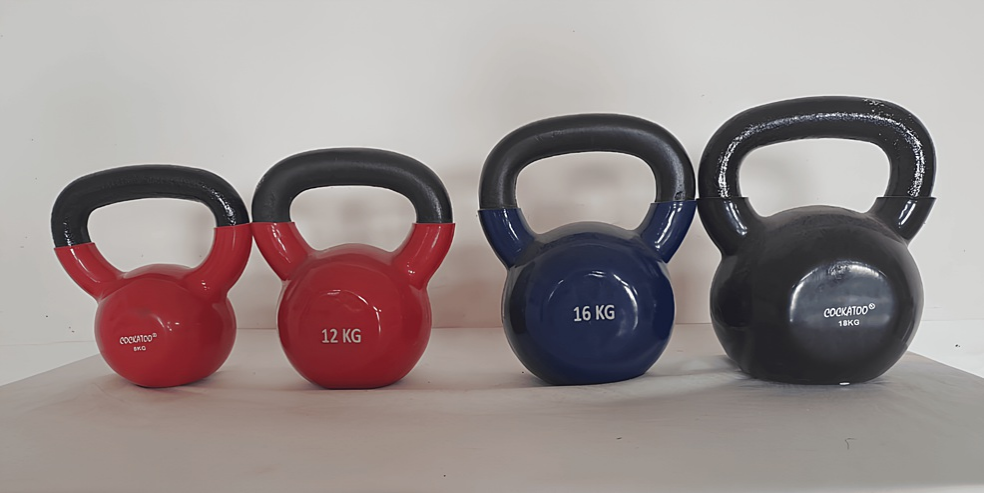
Kettlebells of different weights

Commercial kettlebells (Figure 1) are offered in capacities that vary from 3 pounds to 100 pounds (or more) [14]. The weights that are used with kettlebell swings are usually limited to 70 pounds and are determined on the basis of a particular relative load [15-19]. The kettlebell has a variety of possible clinical applications, including dynamic flexibility exercises and power training [20]. The invention of George Kessler resulted in a kettlebell with a largely hollow body and an angled handle that is firmly attached to its frame at two locations close to the peak of the body. The handle is reduced in length, with the grasping zone containing the tightest portion of the handle and the handle coupling areas containing the most stretched-out portion. The lowering of the handle improves cross-sectional quality where the handle and body converge [21]. The fact that kettlebell training is fundamentally functional, for example, it integrates a variety of muscle groups into these fundamental swinging or ballistic exercises (which includes quick acceleration and deceleration), contributes to its increasing popularity. These methods require concurrent core stability and induce both concentric and eccentric contraction [15]. Through the swinging movement, the kettlebell's center of mass forms a long lever arm [14]. The handle design enables the user to execute ballistic swaying motions, which may enhance their endurance, strength, and power. Furthermore, in comparison with conventional free-weight grips, there is a belief that the grip which enables the user to execute specific ballistic motions may further improve functional muscle group activation. The fact that kettlebells are more affordable than Olympic weight training equipment adds to their allure [16]. Activities with kettlebells may be a good substitute for barbell exercises in order to create a stronger response and enhance sprinting ability because kettlebells are lightweight and readily transportable.

Additionally, it has been demonstrated that a kettlebell swing with mild loads generates power results that are comparable to jump squats with more weight and start a larger ratio of propulsive forces from the horizontal to the vertical than with barbell and jump squatting exercises [6,15,22,23].

Athletic performance is a multifaceted concept that encompasses strength, power, agility, endurance, and overall physical prowess. Athletes and fitness enthusiasts constantly seek innovative training methods to enhance their performance and achieve their peak potential. Kettlebell training is one such technique that has drawn much interest lately. The present review focused on methodically identifying and assessing the current body of knowledge regarding kettlebell training. The study also focused on the impact of kettlebell training on athlete's strength, power, and endurance.

Objective of the study

Increasing strength, power, and endurance is a constant need in the fields of sports medicine, athletics, rehabilitation, and fitness. There is a continuous trial and adoption of new training techniques with differing levels of outcome analysis. The present review included the different considerations pertaining to the structure and design of resistance training programs. The review further depicts the resistance training progression in the context of each person's unique training status and objectives. The study also addresses some of the key progression ideas that have been suggested and recently published on the efficacy of kettlebell interventions in athletes.

To fully understand the impact of kettlebell training on athletic performance, however, a detailed study of available literature is required. The goal of this review is to examine the various aspects of kettlebell training and how it impacts athletes' power, strength, endurance, and other performance metrics. With a thorough examination and synthesis of the literature, this review seeks to shed light on the benefits and limitations of kettlebell training as an additional strategy for enhancing athletic performance. Trainers, athletes, and fitness experts who wish to maximize their training plans and learn how to use kettlebell exercises to their fullest potential will find it to be an invaluable tool.

The topic of discussion centers on an intriguing query: to what extent does kettlebell training impact sports performance? Can this seemingly small piece of equipment affect an athlete's ability to perform at their best? This review will look at existing literature to find out if kettlebell training has any potential effects on significant performance indicators, such as strength, power, agility, and injury prevention. The discussion's central question is this: how much does kettlebell training affect athletes' performance?

## Review

Methodology

Data Sources and Search Engines

A search was conducted on electronic databases (Cochrane Library, PEDro, PubMed, Google Scholar) from September 2013 to September 2023, using search terms "kettlebell," "training," "athletes," "players," and "sports" in the title or abstract. The filter for study design, publication type, or limitations of language was applied for the search. Replica documents were eliminated.

Methodology

Study selection: Individually, two reviewers looked through relevant titles and conducted database searches. Titles and abstracts that mentioned the use of kettlebell training as at least one intervention were relevant. The same two reviewers also evaluated the trials that were included after the abstracts were chosen. A third reviewer examined the problem to determine whether there were any variations in the methodological scores.

Eligibility Criteria

Inclusion criteria: The inclusion criteria were randomized controlled trials (RCTs), non-randomized trials, or pre-test/post-test studies that investigated at least one objective strength, endurance, or power performance measure and included at least one kettlebell training intervention in human adults with a control or other form of exercise comparison group, published in English from peer-reviewed journals.

Exclusion criteria: Exclusion criteria were studies that included individuals with significant comorbidities from non-peer-reviewed journals.

Search Strategy

**Figure 2:**
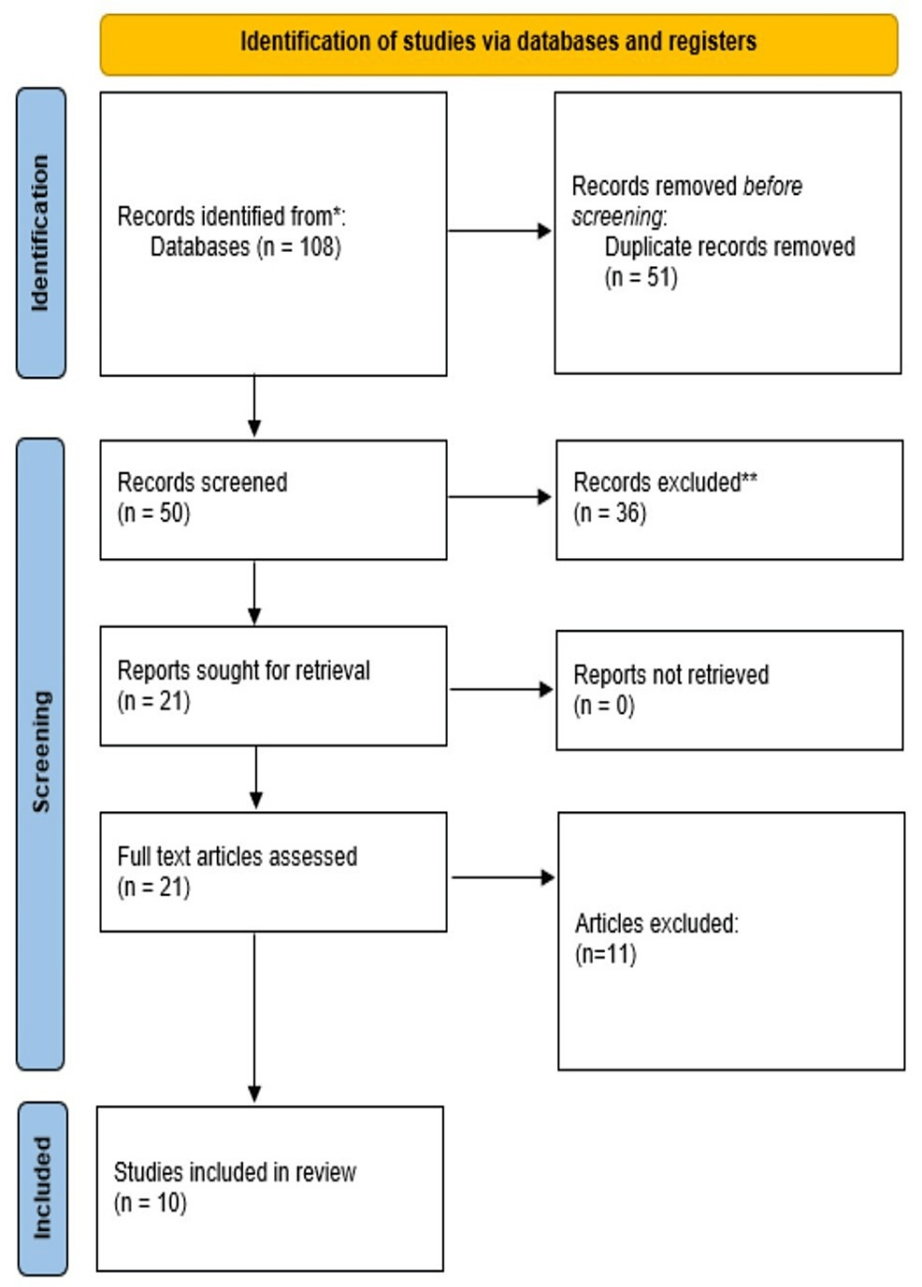
PRISMA flowchart PRISMA: Preferred Reporting Items for Systematic Reviews and Meta-Analyses

There were several research types, such as experimental investigations, RCTs, systematic reviews, and literature reviews. Figure 2 provides an overview of the publications chosen in accordance with the Preferred Reporting Items for Systematic Reviews and Meta-Analyses (PRISMA) recommendations.

Kettlebell training for athletes

Athletes can choose from a large variety of training tools and techniques. Kettlebell practice is one such tool. Kettlebell training has grown in popularity over the last 10 years and is now a respectable choice for conditioning and strength training. Originating in Russia, it is a successful method for boosting aerobic capacity, the strength of the muscles, and the endurance of the muscles as well as decreasing body fat [24]. The kettlebell is frequently compared to a handle-equipped cannonball [25]. Kettlebells are a great tool for full-body, ballistic exercises that require strong muscles, which means that they may help with respiratory and cardiovascular fitness as well as muscle strength [26]. Three methods can be used to implement the concept of progressive overload, which will boost training stress: expanding the volume (number of sets or repetitions) of a training period, increasing the frequency of exercises performed each week, or increasing the load intensity for a training volume. Greater variation in training stimulus can be provided by boosting the load intensity in which progressing the training load which may result in higher adaptations [27].

Applications

Kettlebell training is valuable for building strength, power, and endurance as part of pre-season conditioning programs. Tailored kettlebell exercises address weaknesses and imbalances, aiding in injury prevention and recovery. Kettlebell workouts complement an athlete's primary training regimen, adding variety and preventing overuse injuries. Dynamic kettlebell movements activate muscles and prepare the body for competition when incorporated into pre-game warm-up routines. Execution of 12 minutes of continuous kettlebell swings showed a metabolic challenge of 87% of maximal heart rate and 65% of maximal oxygen consumption to upsurge aerobic capacity with advances larger than that seen with conventional circuit weight training [24]. Heart rate and consumption of oxygen during several five- to seven-minute cycles of self-selected kettlebell workouts were comparatively more to aerobic exercise methods like incline walking, stationary cycling, and running [28]. High-intensity interval-based kettlebell training showed improved grip strength [29].

Training Techniques

**Figure 3:**
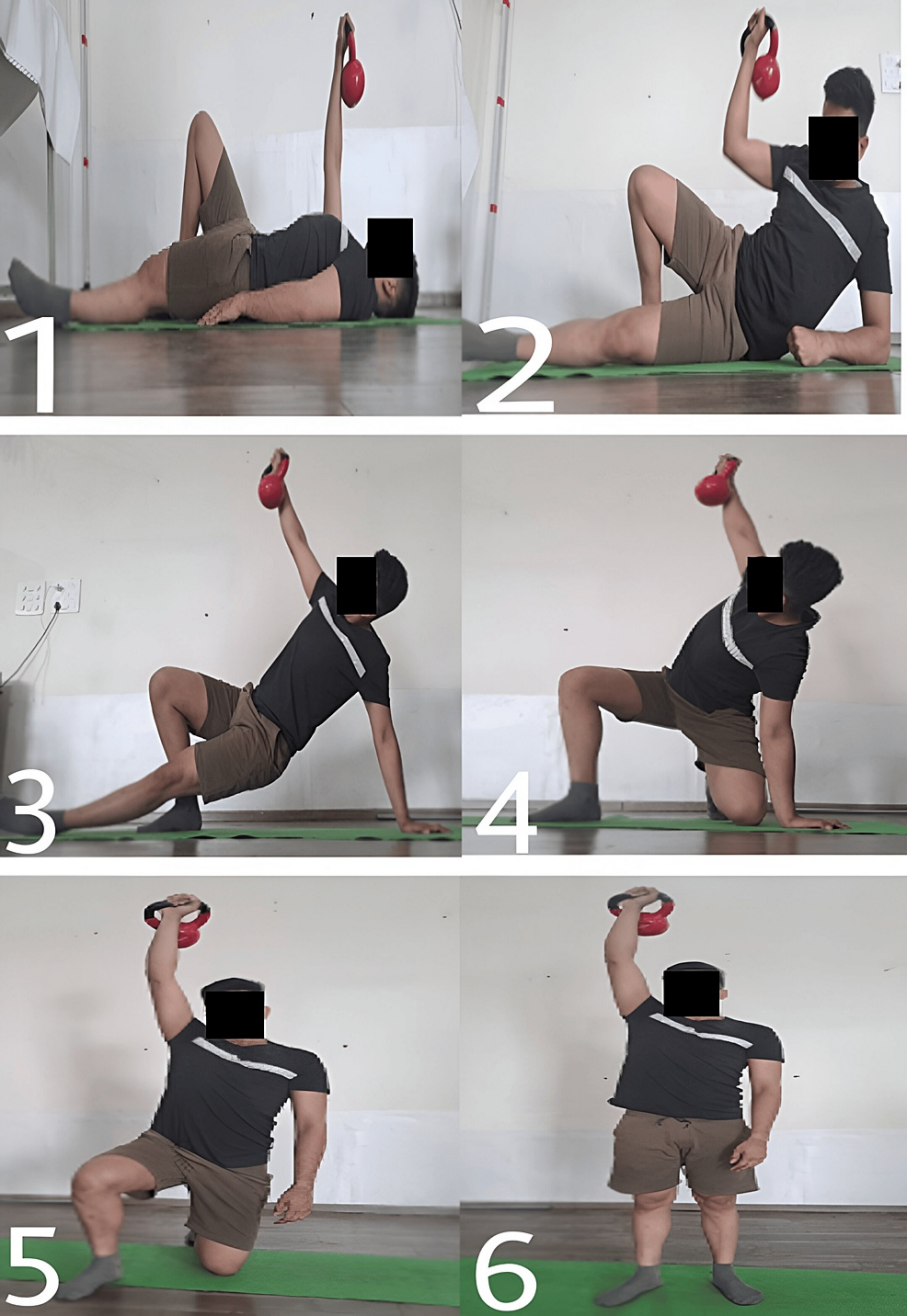
Sequence of the Turkish get-up

**Figure 4:**
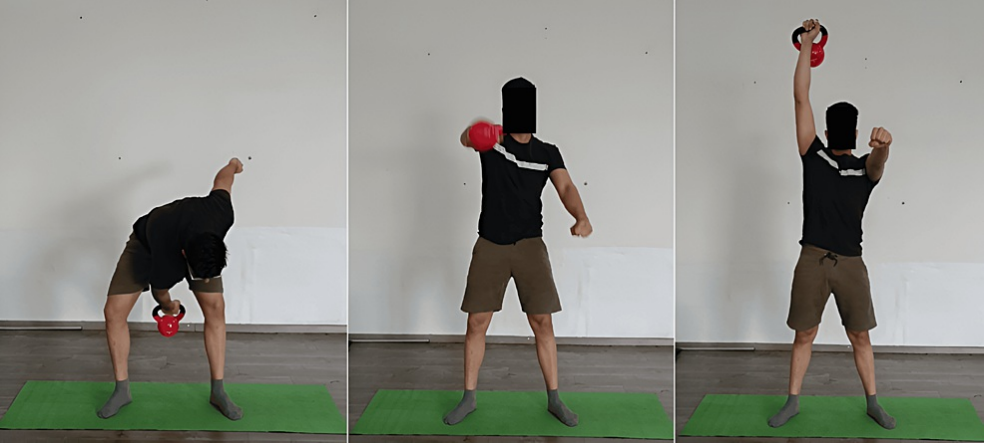
Kettlebell snatch

**Figure 5:**
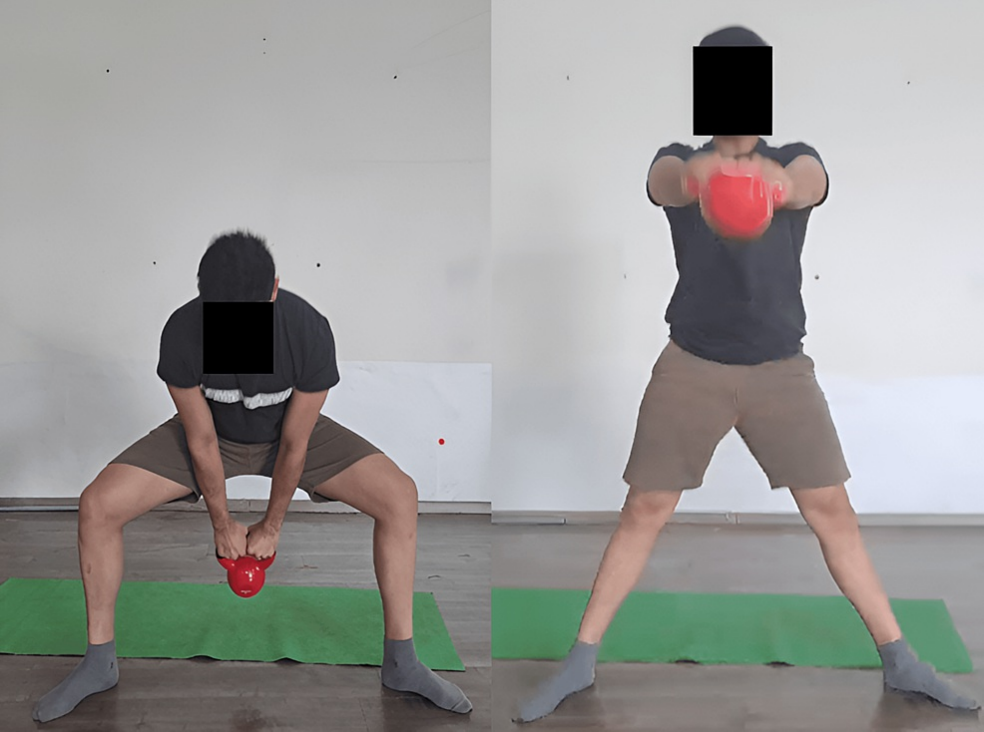
Kettlebell swing

**Figure 6:**
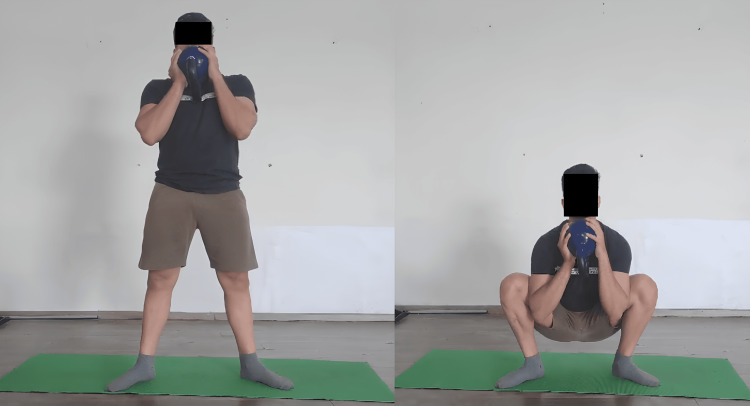
Kettlebell goblet squat

When adjusted and overseen by a sports physical therapist, the kettlebell swing can be an effective rehabilitation tool for patients. It concentrates on controlled hip hinge motions, which strengthen the muscles of the lower back and posterior chain, which are frequently impacted by injuries. The Turkish get-up (Figure 3) is a way that sports physiotherapists can use to increase general stability and mobility. It is a multi-joint, intricate exercise that requires a series of purposeful actions to go from lying down to a standing position while sustaining an overhead kettlebell in a static position. This workout works over a variety of muscle groups and calls for mobility, stability, and coordination. This exercise helps patients who have joint instability or mobility issues recover by incorporating controlled transitions between positions. In the fast-paced exercise known as the snatch (Figure 4), the kettlebell is raised from the floor to an overhead position in a single, smooth motion. It's a great exercise for developing power because it calls for explosiveness and coordination. Lifting the kettlebell to the shoulder (clean) and then pressing it overhead (jerk) is the exercise. This intricate exercise improves the power, strength, and coordination of the upper body. Irrespective of the swing or snatch style, the kettlebell swing produces a hip hinge squat motion sequence in addition to a series of quick, significant muscle activation-relaxation phases. Because of this, this particular exercise might be a great fit for some workout regimens that emphasize the advancement of posterior chain power around the hip. On the other hand, this workout produces distinct shear and compressing load ratios over the lumbar spine. The additional painless advantages of kettlebell swing require shear stability and tolerance to posterior shear loading. The quantitative analysis thus reveals the widespread belief that kettlebell swings (Figure 5) can improve and restore back functioning and fitness [30]. The goblet squat (Figure 6) is a squat that requires holding a kettlebell close to the chest. It promotes the growth of core engagement, stability, and leg strength.

Kettlebell training offers a comprehensive array of favours for athletes. The jumping height of the kettlebell training group increased significantly by 1.5 cm between the baseline and follow-up [31]. The subjects experienced an increase in clean and jerk strength of about 4 kg (10%) and bench press strength of about 13 kg (30%) [32]. It enhances functional strength and power by activating the kinetic chain and improving neuromuscular coordination, making it crucial for injury recovery and prevention. Dynamic kettlebell exercises like Turkish get-ups foster joint mobility and flexibility of athletes, especially those recovering from surgeries. Core stabilization and rehabilitation are prominent aspects, focusing on core strength, vital for athletes recuperating from back or core injuries. Incorporating high-intensity interval training within kettlebell workouts enhances cardiovascular fitness, serving the recovery needs of athletes. Furthermore, kettlebell training emphasizes functional movement patterns, enabling patients to regain the ability to perform daily and sport-specific activities with increased effectiveness and safety [33].

When engaging in kettlebell training, potential risks and considerations must be carefully managed. Incorrect form may result in injuries, particularly in the lower back and shoulders, underscoring the importance of proper instruction and supervision. Overtraining, without adequate rest, can lead to overuse injuries, necessitating a balanced inclusion of kettlebell workouts within an athlete's overall training regimen.

Inadequate warm-up routines heighten the risk of injury, emphasizing the need for pre-session mobility exercises and dynamic stretches. Selecting an appropriate kettlebell weight is pivotal, as starting with excessive weight can compromise form safety; instead, gradual progression in weight should align with an athlete's increasing strength. A summary of the articles reviewed has been mentioned in Table 1.

**Table 1:** Summary of articles reviewed for kettlebell training RCT: randomized controlled trial; BMD: bone mineral density; EMG: electromyography; PPT: pressure pain threshold; SD: standard deviation; HIIT: high-intensity interval training

Sr. no.	Author and year	Study design	Participants	Intervention	Outcome	Conclusion
1	Melo et al. (2023) [34]	Quasi-experimental study	18 young (12-17-year-old) female artistic gymnasts	Kettlebell swing training and regular kettlebell training	Maturity, cardiorespiratory fitness, capillary blood lactate	When it comes to improving the cardiorespiratory and metabolic demands as well as recovering kinetics during a simulated competition of female artistic gymnastics, the effectiveness of claims associated with kettlebell activity is not evident. The chosen load, swing cadence, duration, and usual+kettlebell protocol's lack of specificity are all potential causes
2	Ooraniyan et al. (2022) [35]	RCT	30 handball players aged 21-25 years	Circuit training with kettlebell and control group	Explosive power and strength endurance	Explosive power, strength, and endurance improved by 11% after eight weeks of a circuit training regimen with kettlebells
3	Seethalakshmi and Suresh (2022) [36]	RCT	40 female volleyball players aged 18-25	Kettlebell training and control group	Speed and endurance	In relation to speed, it was revealed that, in the pre test, the mean score was 7.685 and SD value was 0.213, and in the post test, the mean score was 7.674 and SD value was 0.218. Because of the influence of kettlebell training, there was a notable improvement in muscular endurance but not in speed
4	Ahmed et al. (2022) [37]	RCT	30 under 12 soccer players	Kettlebell training	BMD and certain skillful variables	Kettlebell training helped to improve physical and skillful variables like push-pull leg by 15%, barbell bench by 11%, and bone density by 17% for children in soccer
5	Ullah et al. (2021) [38]	Experimental research study	60 volunteer amateur players	Kettlebell vs. battle rope	Explosive power	Both kettlebell and battle rope HIIT protocols are good for explosive power improvement. Significant improvement in explosive power with both kettlebell (13.73±2.22 vs. 21.31±1.71) and battle rope (13.95±2.03 vs. 18.90±1.08) training was noted
6	Andersen et al. (2019) [39]	Cross-over study	50 healthy men	Kettlebell training	EMG	The erector spinae activation during the one-armed swing was 14-25% higher on the contralateral compared to the ipsilateral side in both exercises. Further, the contralateral side was 14% more activated during the two-armed swing compared to the ipsilateral side during the one-armed swing. For the rectus abdominis muscle, the two-armed swing induced higher activation of the rectus abdominis compared to the one-armed swing on both the contralateral side 40% and ipsilateral side 59%. There were no differences for the external oblique muscle
7	Seethalakshi and Suresh (2019) [26]	RCT	30 collegiate women's volleyball players	Kettlebell training	Explosive strength and strength endurance	Kettlebell training boosted the subjects' explosive strength by 9% and strength endurance by 5%
8	Elumalai and Kannan (2019) [21]	RCT	30 inter-collegiate-level badminton player	Kettlebell and Swiss ball training	Short service and long service	Swiss ball training showed a notable improvement of 8% in short service, while the kettlebell training group showed a noteworthy improvement of 7% in long service
9	Keilman et al. (2017) [40]	Two-armed RCT	60 healthy athletes	Kettlebell swings	PPT	Pressure algometry evaluation of the PPT results in a decrease in muscle sensitivity to noxious stresses
10	Jay et al. (2013) [31]	RCT	40 healthy athletes	Kettlebell training	Postural coordination and jump performance	Advantages from collaborative kettlebell training include improved overall health, job satisfaction, and self-reported muscle strength which are all important psychosocial variables

Discussion

The current review is the first attempt to integrate all presently available studies on the effectiveness of kettlebell training programs in many athletes or sports professionals. According to the research synthesis, it is suggestive that kettlebell training has been effective in enhancing strength and power in athletes. Numerous studies have demonstrated significant improvements in maximal strength, power output, and muscular endurance when incorporating kettlebell exercises into training regimens in athletes.

In addition to being a cost-effective substitute for those without access to gyms and weight rooms, kettlebell training has been shown to enhance psychological well-being and quality of life. It is vital to note that other types of physical activity, such as strength, circuit, concurrent, and primarily aerobic training (with a mix of anaerobic and aerobic periods), have been shown to enhance the quality of life and health status-related parameters in clinical and healthy populations, despite their differences in biomechanics and technical aspects from kettlebell exercise [41]. Research by McGill and Marshall indicates that when it comes to loading patterns, the kettlebell swing exercise might be different from conventional weight training lifting. He discovered that swinging a kettlebell may cause the L4 vertebrae on L5 to shear posteriorly, as opposed to a traditional lift, which causes a bias towards anterior shear. This mechanism could contribute to the benefits of back pain reduction [30].

Even with all of the advantages that kettlebell training offers athletes, more investigation and study are still needed. Future research can look into the best training plans, how programs are put together, and how kettlebell training affects various athletic groups over the long run. Furthermore, contrasting kettlebell workout with other conditioning and strength-building techniques might offer insightful information about its relative effectiveness.

## Conclusions

To sum up, kettlebell training is a flexible and useful strategy for improving players' performance in a variety of sports. It adds value to athlete training programs by enhancing strength, power, endurance, explosive power, and postural coordination. But it is important to recognize that kettlebell training should be carefully included and in line with the individual goals and needs of the athlete. More studies in this area will help us better understand how to get the most out of kettlebell training for athletes, which will result in more specialized and efficient training plans.
